# Chromogranin A-Associated Tubulopathy in a Patient With Neuroendocrine Tumor

**DOI:** 10.7759/cureus.99048

**Published:** 2025-12-12

**Authors:** Matthew Sanborn, Alisha Maity, Vladimir Limonnik, Aqeel Siddiqui, Steven Salvatore, Terri McHugh

**Affiliations:** 1 Internal Medicine, Lankenau Medical Center, Wynnewood, USA; 2 Hematology Oncology, Lankenau Medical Center, Wynnewood, USA; 3 Oncology, Mainline Health, Philadelphia, USA; 4 Hematology Oncology, Thomas Jefferson University Hospital, Sidney Kimmel Cancer Center, Philadelphia, USA; 5 Nephrology, Lankenau Medical Center, Wynnewood, USA; 6 Pathology and Laboratory Medicine, Weill Cornell Medicine, New York, USA

**Keywords:** acute tubular necrosis, chromogranin a, neuroendocrine tumor, renal dysfunction, tubulopathy

## Abstract

Chromogranin A (CGA) is a peptide secreted by neuroendocrine cells and filtered by the kidneys. Excessive CGA secretion is common in neuroendocrine tumors (NETs) and has been linked to organ dysfunction, though direct kidney injury remains rarely described.

We present a woman in her mid-70s diagnosed with metastatic, well-differentiated pancreatic NET who developed progressive renal dysfunction. Initial CGA levels exceeded 86,000 ng/mL and later rose to >500,000 ng/mL. Despite the absence of nephrotoxic exposures, imaging abnormalities, or significant proteinuria, her serum creatinine increased from a baseline of 1.0 mg/dL to 5.6 mg/dL over several months, necessitating hemodialysis. Renal biopsy revealed acute tubular injury characterized by prominent intracytoplasmic granules in tubular epithelial cells, which were strongly positive for CGA on immunohistochemical staining, without evidence of immune complex disease. These findings suggested CGA-induced tubulopathy as the cause of her acute tubular necrosis (ATN). The patient’s NET demonstrated partial radiologic response to treatment with octreotide and everolimus, but she remained dialysis-dependent until her passing six months later.

This case highlights CGA tubulopathy as a rare but significant cause of acute renal failure in patients with NETs. It emphasizes the importance of early nephrology involvement and proactive renal monitoring in patients with markedly elevated CGA levels. Further research is needed to elucidate the mechanisms of CGA-induced nephrotoxicity and the potential reversibility of renal injury with NET-directed therapies.

## Introduction

Chromogranin A (CGA) is a peptide secreted by endocrine and neuroendocrine cells, filtered by the glomerulus, and secreted in excess by neuroendocrine neoplasms. Clinical presentations of neuroendocrine tumors (NET) vary widely, ranging from vague abdominal discomfort to pronounced hormone-related syndromes, which often delay diagnosis. We present a patient with a known NET who developed acute renal dysfunction, potentially linked to CGA secretion. Despite the absence of nephrotoxic agents, normal imaging findings throughout her hospitalizations, and an unrevealing serologic workup, the patient’s renal function progressively deteriorated, ultimately requiring dialysis. Renal biopsy revealed acute tubular necrosis (ATN) with intratubular accumulation of chromogranin. This case suggests a potential connection between NET CGA secretion and ATN, resulting in significant acute renal dysfunction.

Neuroendocrine tumors (NETs) are a heterogeneous group of neoplasms that arise from neuroendocrine cells. The most common primary sites include the gastrointestinal tract (particularly the small intestine, appendix, and rectum), pancreas, and bronchopulmonary system [1]. The incidence of NETs in the United States has risen over the past decade, from approximately 1.6 per 100,000 in the 1970s to nearly 8.5 per 100,000 by 2021 [2]. A key feature of NETs is their ability to secrete peptide hormones and neuroamines, giving rise to specific circulating biomarkers. Among these, CGA is the most widely used. CGA is a secretory granule protein co-released with hormones, and elevated levels correlate with tumor burden and progression [3] as well as poorer prognosis in NET patients [4]. In rare instances, markedly elevated CGA may accumulate within renal tubules, and diagnosis of CGA-associated nephropathy is confirmed by renal biopsy showing tubular injury with CGA-positive intracytoplasmic inclusions in the absence of other causes of renal damage.

NETs are characterized by their origin, stage, and histological characteristics, and secrete CGA, which is known to cause organ dysfunction [5]. NET-related symptoms are related to hormone hypersecretion. Korse et al. demonstrated that elevated CGA levels in patients with NETs and carcinoid heart disease are associated with decreased overall survival [4]. While elevated CGA levels in NETs are linked to renal dysfunction, the literature on severe acute kidney injury caused by CGA is limited. We present a patient with a NET who developed significant renal dysfunction, requiring dialysis, with a renal biopsy consistent with ATN. This case underscores the importance of recognizing NET-related renal complications in patients with unexplained renal impairment.

## Case presentation

**Figure 1:**
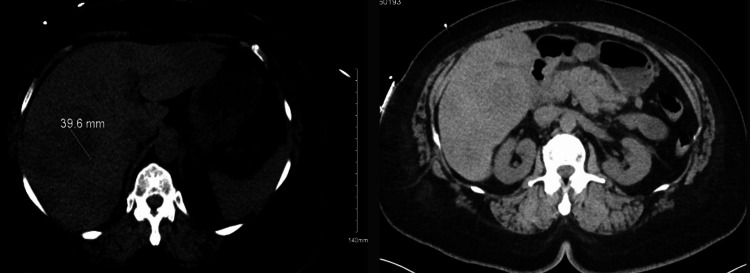
CT Abdomen Pelvis Extensive liver masses suspicious for metastatic disease and suggested enlargement of the pancreatic body, which may reflect a neoplasm or metastasis.

A mid-70s female initially presented to the ED with abdominal discomfort, nausea, decreased oral intake, and unintentional 20-30-pound weight loss. Further questioning revealed the patient has had this abdominal discomfort for around a month. On exam, the patient was not in acute distress; however, they did have dry mucous membranes. Cardiac and pulmonary exams were unremarkable. The patient had no abdominal tenderness, no palpable masses, no abdominal distension, and no jaundice. CT abdomen/pelvic showed extensive liver masses, suspicious for metastatic disease, enlargement of the pancreatic body, and mild thickening of the left adrenal gland. CT chest showed nonspecific 1-2 mm pulmonary nodules in the left upper lobe. Key initial labs are outlined in Table 1, and the CT abdomen pelvis is represented in Figure 1.

**Table 1:** Initial Labs

Test	Patient Value	Reference Range
Creatinine	1.7 mg/dL (baseline 1.0 mg/dL)	0.6 – 1.2 mg/dL
AST	56 IU/L	13 – 39 IU/L
ALT	16 IU/L	7 – 52 IU/L
Alkaline phosphatase	130 IU/L	34 – 125 IU/L
Total bilirubin	0.2 mg/dL	0.3 – 1.2 mg/dL
CEA	0.7 ng/mL	< 3.0 (non-smokers)
AFP	206 ng/mL	<= 9/0 ng/mL
CA 19-9	<0.8 U/mL	< 35.0 U/mL
Chromogranin A (initial)	86,549 ng/mL	< 101.8 ng/mL
Serum creatinine (baseline)	1.0 mg/dL	0.6 – 1.2 mg/dL

**Figure 2:**
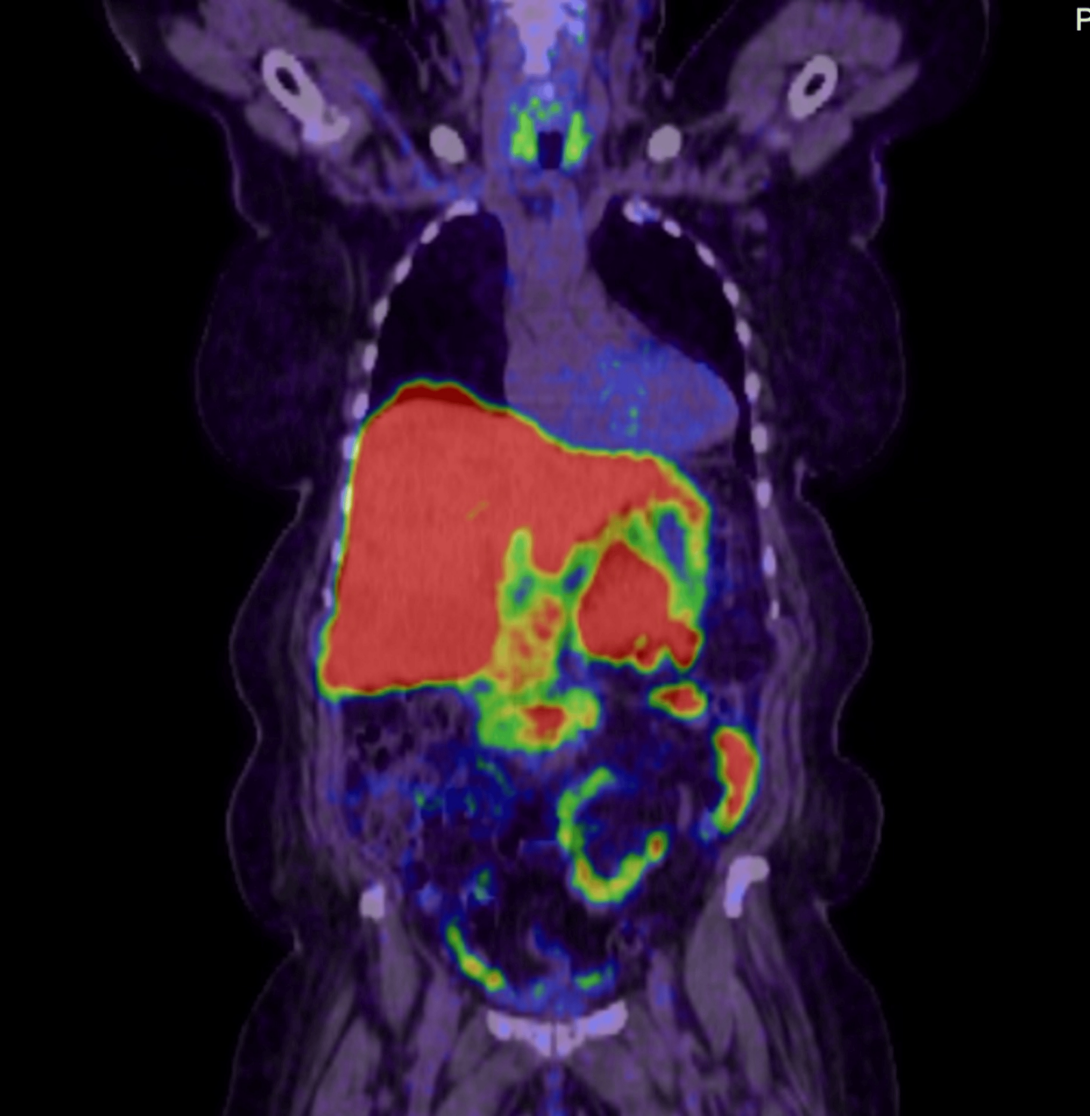
CT PET NETSPOT Approximate size of pancreatic mass is 5.9 × 3.3 cm. The largest mass in the liver is located in the inferior right hepatic lobe, measuring 5.6 × 5.6 cm. Somatostatin-positive pancreatic mass and numerous hepatic metastases. No additional sites of disease were identified.

Liver biopsy showed a metastatic, well-differentiated neuroendocrine tumor, grade 2, positive for pancytokeratin, CD56, CGA, and INSM1, negative for arginase, glypican-3, and HepPar 1. CT PET NET SPOT, a somatostatin receptor-based PET/CT used to detect neuroendocrine tumors, 3 weeks later showed a somatostatin-positive pancreatic mass with numerous hepatic metastases (Figure 2). Initial CGA at this time was 86,549 ng/mL. Given a clinically significant tumor burden and positive somatostatin, octreotide LAR 30 mg IM every 28 days was started.

The patient then presented to the nephrology office 3 months later as part of routine follow-up in the setting of being found with a serum creatinine of 2.4 on outpatient labs, increased from a baseline of 1 mg/dL 6 months prior. No nephrotoxic agents, including prescription or over-the-counter medications, were identified at the time of evaluation. A thorough serologic workup, including antinuclear antibodies (ANA), complement levels, hepatitis C panel, hepatitis B panel, antineutrophil cytoplasmic antibodies (ANCA), rapid plasma reagin (RPR), anti-glomerular basement membrane (anti-GBM), anti-streptolysin O (ASO) titer, and HIV, was negative. Table 2 contains key laboratory results. Renal ultrasound demonstrated well-appearing kidneys with normal cortical echogenicity without hydronephrosis. The differential diagnosis for progressive renal dysfunction included obstructive uropathy, immune-mediated glomerulonephritis, and other causes of tubular injury; however, these were considered unlikely given normal renal imaging, negative serologic evaluation, and absence of nephrotoxic exposures.

**Table 2:** Additional Labs

Test	Patient Value	Reference Range
Urine microalbumin/Cr ratio	119.9 ug/mg	< 30 ug/mg
IgG kappa free light chain	209 mg/L	8.96 – 34.28 mg/L
IgG lambda light chain	35.8 mg/L	5.7 – 26.3 mg/L
Kappa: Lambda ratio	5.8	0.64-1.83

The patient was hospitalized 3 months later with a creatinine of 3.7 mg/dL, which improved to 3.5 mg/dL with hydration. Renal ultrasound during this admission was negative for renal artery stenosis or renal vein thrombosis. Two weeks later, the patient was admitted again with worsening renal function, presenting with a creatinine of 4.2 mg/dL and clinical volume overload. Bumetanide was initiated but discontinued due to further creatinine increase to 5.6 mg/dL. Hemodialysis was eventually initiated during this hospitalization due to progressively worsening renal function. The patient underwent a renal biopsy during this admission, at which time the CGA level was >500,000 ng/mL.

**Figure 3:**
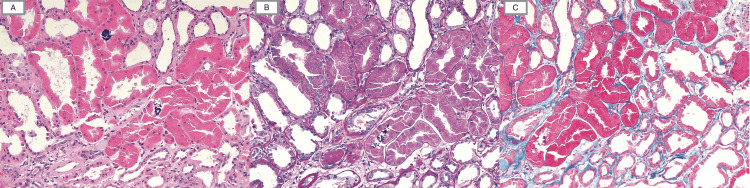
Light Microscopic Appearance of Renal Tubules With Eosinophilic and PAS-Positive Granules Light microscopic appearance of renal tubules, which are distended by foamy cytoplasm, including eosinophilic, PAS-positive, and fuscinophilic granules within their cytoplasm. Occasional intraluminal calcium phosphate calcifications are also present. (H&E (A), PAS (B), and Trichrome (C) stains, 20×)

**Figure 4:**
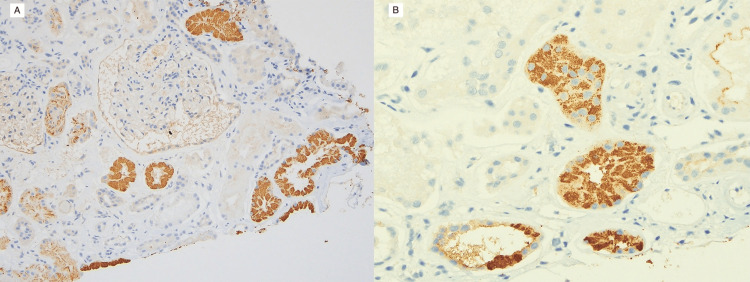
Chromogranin A Immunohistochemical Staining of Proximal Tubules Chromogranin A immunohistochemical stain showing strong positivity within the proximal tubules’ cytoplasm (20× (A), 40× (B))

The renal biopsy showed acute tubular injury with prominent intracytoplasmic granules within the tubular epithelial cells that are eosinophilic on H&E, PAS weakly positive, and fuscinophilic by trichrome staining (Figure 3). The intratubular granules were strongly positive for chromogranin A by immunohistochemical staining (Figure 4). The glomeruli were histologically unremarkable, with global glomerulosclerosis of 10 of 23 glomeruli. There was moderate interstitial fibrosis and tubular atrophy, along with moderate arteriosclerosis and arteriolosclerosis. No evidence of immune complex-related glomerular disease was found. These findings confirmed that the tubules contained significant resorption of chromogranin, likely leading to the acute tubular injury, as a cause for the patient’s ongoing renal dysfunction.

The patient also underwent a repeat liver biopsy during this admission, which showed pathology findings similar to the initial liver biopsy. PET/CT showed a hypermetabolic liver and pancreatic mass consistent with known disease; however, there was an overall increase in size, suggesting progressive disease. As such, everolimus was added to her treatment regimen.

A follow-up PET/CT scan three months later showed a partial response to therapy, with a significant decrease in the size and metabolic activity of multiple liver metastases. However, there was an interval development of left lower lobe nodular opacities, possibly representing pulmonary metastases. Throughout this period, the patient remained on hemodialysis due to renal failure. The patient’s CGA level at this time had decreased to 162,756 ng/mL (reference range: < 101.8 ng/mL).

## Discussion

CGA is a peptide secreted by endocrine and neuroendocrine cells. It is often secreted in excess in neuroendocrine neoplasms. The glomerulus filters CGA and can be detected in the urine.

NETs are a diverse group of neoplasms arising from neuroendocrine cells with nerve and endocrine cell characteristics. Classification is determined by differentiation and grade, with well-differentiated classified as grades 1 or 2 and poorly differentiated classified as grade 3. NETs can be functional when secreting hormones, leading to clinical syndromes (carcinoid syndrome) or non-functional, in which no active hormones are produced. NETs can originate from various organs, including the gastrointestinal tract, lungs, pancreas, and adrenal glands. Diagnosis is commonly made by biochemical markers (CGA and synaptophysin) and imaging (CT, MRI). NET treatment is dependent on location, grade, and staging, with surgery as an option for primary/localized tumors; somatostatin analogs, targeted therapies (such as everolimus), and chemotherapy are reserved for higher-grade/poorly differentiated tumors [1].

CGA tubulopathy is a rare cause of acute renal failure in patients with neuroendocrine tumors. The literature shows a lack of histologic correlation between CGA levels and direct tubular damage, partly because most of the patients studied did not undergo renal biopsy. A retrospective study by Sekulic et al. described two potential mechanisms of acute tubular injury attributed to CGA [6]. The proposed pathogenesis involves excessive proximal tubular reabsorption of CGA, resulting in lysosomal overload and cytoplasmic accumulation that leads to tubular epithelial injury. Alternatively, CGA may precipitate within the tubular lumen and form an obstructive tubular obstruction and ischemic injury [6].

Our case underscores the importance of early nephrology involvement in patients with NETs secreting CGA who develop kidney injury. At NET diagnosis, we suggest establishing baseline renal function, including creatinine, eGFR, urinalysis, and urine protein excretion. We recommend monitoring renal function every 4-6 weeks, with an increase in frequency if CGA levels are extremely high (>10,000 ng/ml) or rising rapidly, allowing for prompt intervention when necessary. It is also essential to evaluate for symptoms of renal dysfunction at each visit, such as oliguria, edema, hypertension, and fatigue/weakness, as these symptoms can precede laboratory changes. Prompt referral to nephrology allows for advanced investigations, including renal-specific imaging and biopsies, and monitoring for the need for hemodialysis. Moreover, educating patients to recognize the signs of renal impairment can facilitate early detection and intervention.

## Conclusions

Further investigations into the correlation between NETs secreting CGA and renal dysfunction are warranted. There is a paucity of large-scale studies to establish a link between NETs secreting high levels of CGA and renal dysfunction, limiting the ability to generalize findings and draw comprehensive conclusions about the mechanism of CGA-induced nephrotoxicity. Future studies should investigate whether treating the NET, thereby decreasing CGA levels, can help recover renal function. Simultaneously, investigations can also determine whether hemodialysis has a role in recovering renal function for these patients.

CGA secretion from a NET can lead to ATN and cause significant renal dysfunction necessitating dialysis; monitoring guidelines, including baseline renal function labs and clinical assessments of kidney dysfunction, are necessary for these patients, as prompt intervention can lead to better outcomes. More studies are needed to understand the mechanisms by which elevated CGA levels from NETs lead to ATN, as current evidence is limited and predominantly based on isolated case reports.
